# Creative Sparks or Paralysis Traps? The Effects of Contradictions on Creative Processing and Creative Products

**DOI:** 10.3389/fpsyg.2018.01489

**Published:** 2018-08-21

**Authors:** Goran Calic, Sébastien Hélie

**Affiliations:** ^1^DeGroote School of Business, McMaster University, Hamilton, ON, Canada; ^2^Department of Psychological Sciences, Purdue University, West Lafayette, IN, United States

**Keywords:** creativity, paradoxical tensions, computer simulation, individual differences, cognition, thinking style, business management, integrative complexity

## Abstract

Paradoxes are an unavoidable part of work life. The unusualness of attempting to simultaneously satisfy contradictory imperatives can result in creative outcomes that simultaneously satisfy both imperatives by inducing search for, and selection of, novel and useful solutions. Likewise, extant research suggests that paradoxes can also result in anxiety, defensiveness, and persistence of old ways of doing things. However, there is little work attempting to describe how paradoxes affect cognition and when it results in higher or lower creativity. To tackle this issue, a theory of paradoxical creativity is developed. Paradoxical creativity is the attempt by an individual to creatively resolve a contradiction by simultaneously achieving competing demands. The theory is implemented into a computational model and a simulation is used to describe how paradoxes affect creative cognitive process and how these processes in turn result in higher or lower degrees of creativity. The results show that creative output is enhanced when paradoxes have a balanced effect on the cognitive processes responsible for an individual's capacity to search for new information and willingness to tolerate new ideas. Hence, individuals with high baseline levels of creative cognition are more likely to suffer negative creative performance consequences resulting from contradictory demands. For those individuals, contradictory demands may produce more alternatives, which increases uncertainty and time to insight (if insight is ever reached). This suggests that incentives or rewards to resolve contradictions may have the unintentional effect of reducing creative output in some circumstance.

## Introduction

Challenging organizational issues raise paradoxes, which are “contradictory yet interrelated elements that exist simultaneously and persist over time” (Smith and Lewis, 2011, p. 382). As globalization, competition, and environmental complexity increase, so does the intensity with which paradoxes are experienced, such as the growing pressure on organizational managers to be both cooperative and competitive (Brandenburger and Nalebuff, 2011), profitable and charitable (Hahn et al., 2014), and efficient and effective (van Thiel and Leeuw, 2002). How managers respond to the challenges of these paradoxes can determine an organization's fate (Quinn, 1988; Smith and Lewis, 2011). This paper examines some complications of the prevalent strategy to embrace and work through paradox, particularly the absence of theorizing about differences in thinking style, which are stable information processing characteristics of individuals (Schroder et al., 1967; Driver and Streufert, 1969).

Because paradoxes are persistent and unavoidable in organizational life, the suggested management strategy is to embrace and work through them (Andriopoulos and Lewis, 2010; Smith and Lewis, 2011; Lewis et al., 2014; Smith, 2014). Embracing paradox enables superior performance through the mechanism of creativity (Miron-Spektor et al., 2011; Smith and Lewis, 2011), which is defined as the generation of new and useful ideas. Consequently, creativity enables the manager not only to discover solutions to pressing organizational issues, but to continually improve social and economic performance by discovering new opportunities (Nonaka and Toyama, 2002; Anderson et al., 2014).

Despite frequent references in the management literature to the positive effects of working through paradox (e.g., ambidextrous organizations: Tushman and O'Reilly, 2006), idiosyncrasies of the agents remain undeveloped. While there is extensive evidence on the *average* positive effect of paradox on individual creativity (DeFillippi et al., 2007; Martin, 2009; Miron-Spektor et al., 2011), the key problem of understanding whether all individuals react the same to paradox remains unexplored.

Developing a theory of differences in individual responses to paradox seems especially crucial in the face of general evidence that dissimilarities exist in how individuals process information (Proctor and Vu, 2012) and specific evidence that not all individuals experience paradox equally (Lewis, 2000; Casson, 2005; Smith and Tushman, 2005; Smith and Lewis, 2011; Calic and Helie, 2016; Keller et al., 2017). Individuals may feel frustration when confronted with paradoxes or be completely comfortable with them (Huy, 2002; Lüscher and Lewis, 2008) and they may feel paradoxes are impossible to manage or be an easy undertaking (Smith and Berg, 1997). This challenge is addressed using a psychologically-realistic computer simulation of creative cognition.

This study offers three distinct contributions. First, it offers a detailed examination of the cognitive mechanism through which paradox result in superior performance: *creativity*. Second, it advances the understanding of variance in individual responses to paradox. As such, this study offers recommendations for how to manage paradox for superior creative performance. Finally, it adds to earlier work that describes how paradox affects individual creative performance, specifically the cognitive mechanisms that interact with paradox (Quinn, 1988; Smith and Tushman, 2005; Miron-Spektor et al., 2011; Smith, 2014).

## Current perspectives on organizational paradoxes and creativity

How does working through paradox enhance creativity? Working through paradox points to possibilities. In his studies, Rothenberg (1979) found that creative genius stemmed from the ability to juxtapose opposing ideas. For instance, in science, Einstein's theory of relatively emerged from the juxtaposition of thinking about the same object simultaneously in motion and at rest. In music, Wolfgang Amadeus Mozart's Symphony No. 40 in G Minor illustrates the simultaneous operation of the antithesis of chromaticism (key relationships) and diatonicism (tonic-dominant relationships involving seven tones in the major and minor). In art, Picasso's paintings reflect both calm and chaos. Yet, not all individuals experience tensions raised by paradox equally. An individual's response to paradox will depend on her own cognitive predispositions (Huy, 2002; Lüscher and Lewis, 2008). For instance, an individual may feel a paradox is frustrating or impossible to solve or be a mundane challenge of everyday life (Smith and Berg, 1997).

Smith and Lewis (2011) suggest that paradoxical tensions persist as latent tensions. Latent tensions are contradictions unnoticed by individuals. Such tensions are dormant, unperceived, or ignored, until they are accentuated by environmental factors. Scarcity of resources, plurality of views, or change can render latent tensions salient (Smith and Lewis, 2011). Once salient, tensions become recognized by individuals as paradoxes. In organizational life, globalization may render salient the tensions between diverging viewpoints (Bradach, 1997); technological innovation may render salient the tension between building up or destroying the past to create the future (Andriopoulos and Lewis, 2009, 2010); and hypercompetitive environments may render salient the contradictory demands on scarce resources (Smith and Tushman, 2005). Relying on cognitive tuning theory (Schwarz, 1989, 2002), scholars have argued that the perception of an organizational paradox signals that the individual finds herself in an environment that is unusually complex. This results in a feeling of conflict and a more integratively complex thinking strategy.

In a laboratory study of the effect of paradox on creative performance, operationalized as the number of solutions to a remote associate test (RAT), Miron-Spektor et al. (2011) show that the positive effect of paradoxical tensions on creativity is moderated by a sense of conflict and integrative complexity, a psychological measure that refers to an individual's ability to think about the world in a more complex manner and thus discover new ideas (Schroder et al., 1967; Simon, 1978). In this study, individuals who were primed with paradox experienced an increased sense of conflict and behaved in a more integrative complex manner. However, integrative complexity and a sense of conflict are not purely mutable traits. Individuals have different baselines on these characteristics.

The aforementioned study did not explore whether the stable portion in these individual traits mattered. For instance, would individuals with higher or lower integrative complexity benefit more or would they benefit less from working through paradoxes? The widely held view of integrative complexity is that the more, the better (Perry, 1970; Loevinger and Blasi, 1976; McAdams, 1990). However, evidence from studies of MBA students (Tetlock et al., 1993) and managers (Streufert and Swezey, 1986) suggests that too much integrative complexity can result in excessive anxiety, procrastination, and difficulty in making decisions. What this means for the relationship between organizational paradox and creativity is largely unknown. To answer this question, the current study relies on a psychologically-realistic computer simulation of the explicit-implicit interaction theory (EII) (Hélie and Sun, 2010), an integrative theory of creativity.

## A psychologically—realistic simulation

The current article seeks to utilize a computer simulation to examine the effect of paradox on creative output, measured as the number of ideas that are both *generated and output*. We choose creative output because creative fluency, the number of creative ideas given on any one divergent thinking exercise (Runco and Sakamoto, 1999), is a frequently used measure of individual creativity and highly correlated with originality of ideas [e.g., *r* = 0.81 in Diehl and Stroebe (1987); *r* = 0.69 in Parnes and Meadow (1959)]. This measure is, in itself, agnostic about the originality of the idea. Nevertheless, in the current simulation it serves as a good measure of the creative process simulated by EII.

Because of the critical role of creativity as a mediator between paradox and superior organizational performance (Smith and Lewis, 2011), paradox theory can benefit a great deal from a detailed understanding of creative cognition, including the detailed processes and mechanisms responsible for idea generation and selection. Some of these processes have been tackled with the use of computational modeling and simulations of cognitive architectures (Sun, 2002, 2014; Dollinger, 2011). A cognitive architecture specifies the essential mechanisms, structures, and processes in the form of a domain-generic computational model. Such a model can be used for a broad analysis of cognition and behavior (Sun, 2006). The function of a cognitive simulation is to provide an infrastructure that enables a deeper understanding of various components and processes of the mind. In this way, a simulation serves as the initial set of assumptions to be used for further theory development (Sun and Hélie, 2015).

Computer simulations provide several advantages. First, the use of simulated agents allows for direct modulation of integrative complexity and the observation of its effect on creativity. Second, computer simulations allow for seeding all agents with the same knowledge. Therefore, if an agent is not responding creatively, it is not because it is less knowledgeable or experienced than other agents (Taylor and Greve, 2006). Third, the interactions and outcomes of otherwise private cognitive processes can be observed (Sun and Hélie, 2015). Although this work constitutes the first attempt to use psychologically-realistic agents to extend the paradox literature, simulated agents have already been used to account for similarly complex phenomena, such as research output in the scientific community and organizational decision-making (Sun and Naveh, 2004).

### Theoretical perspective: explicit-implicit interaction theory

A more fine-grained understanding of the relationship between paradoxical tensions and creativity can be gained from using a simulation model that describes creativity based on EII theory (Hélie and Sun, 2010). Explicit-implicit interaction theory integrates many existing theories of creative problem solving, such as theories of incubation (e.g., unconscious work theory, conscious work theory, recovery from fatigue, forgetting of inappropriate mental sets, remote association, opportunities assimilation), insight (e.g., constraint theory, fixation theory, associationistic theory, evolutionary theory), and creativity (e.g., Geneplore, evolutionary theory of creativity). Even though EII theory is a high-level domain generic model of creative cognition, it nonetheless suggests process-based explanations that are sufficiently detailed for implementation using a computer model.

#### Five principles of EII theory

Explicit-implicit interaction theory relies on five basic principles (Hélie and Sun, 2010). The first principle is the existence of explicit and implicit knowledge and processing (Sun, 2002). Explicit processes are consciously available and perform some form of rule-based reasoning satisfying some relatively crisp and exact conditions. In contrast, implicit processing is not consciously available, and satisfies soft conditions using “associative” processing. Second, explicit knowledge and implicit knowledge are often “redundant”: although they are represented differently, they may contain the same knowledge. For instance, consider the similarities and differences between the explicit knowledge of how to perform a tennis serve vs. the implicit skill of performing the serve. Third, explicit and implicit processes are invoked simultaneously in most tasks under most circumstances. As such, both processes can end up with compatible or conflicting conclusions that contribute to the overall output. Fourth, the results of explicit and implicit processing are integrated when generating ideas. As a result, no task is purely explicit or implicit. Instead, the “explicitness” or “implicitness” of a task lies on a continuum. Fifth, processing is often iterative and potentially bidirectional between implicit and explicit processing. If the integrated outcome of explicit and implicit processing does not yield a definitive result, which is a result in which one is highly confident, and if the time constraint has not been met, another round of processing may occur.

#### Four stages of EII theory

**Figure 1:**
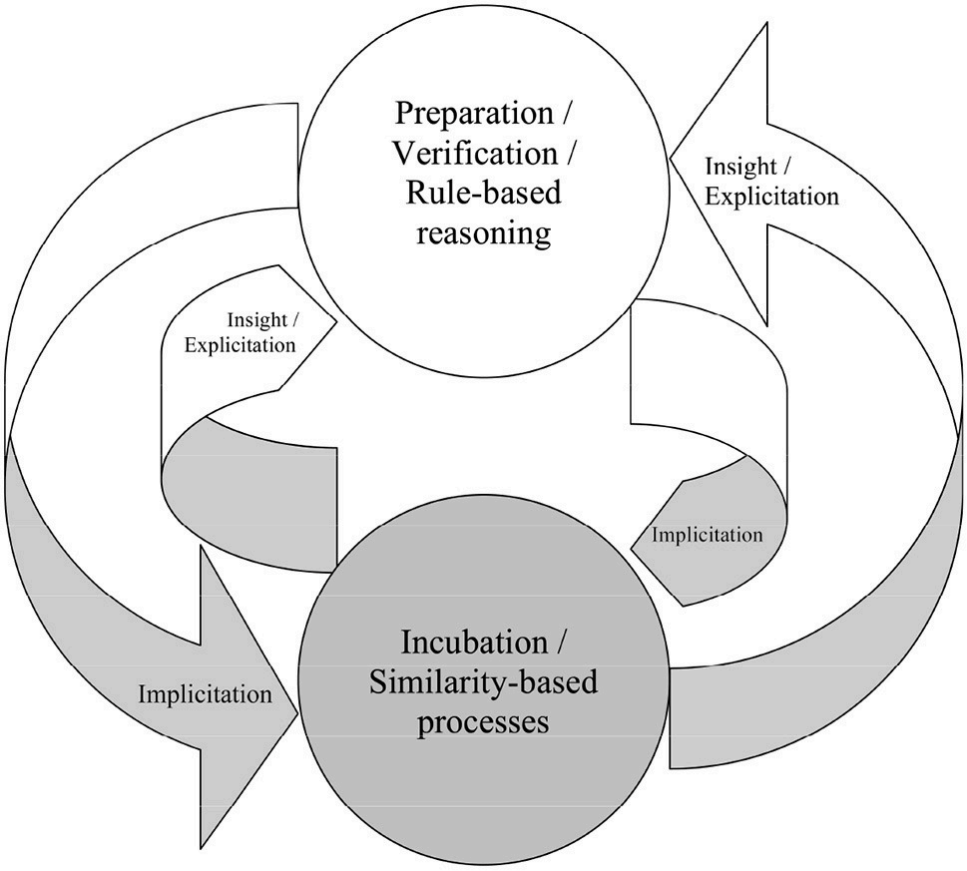
Information flow in the EII theory. The gray sections are implicit while the white sections are explicit.

The preceding assumptions allow for a conceptual model that captures creativity according to Wallas's (1926) analysis of creative problem solving (see Figure 1). Wallas's first stage of creative problem solving is the preparation stage. Wallas described the preparation stage as involving logic and reason. This is captured by explicit processing in EII theory. Explicit knowledge is usually rule-based, which includes logic-based reasoning as a special case. Also, the preparation stage has to be explicit in EII because people are responding to explicit verbal instructions, forming representations of the problem, and setting goals.

The next stage, incubation, happens when an impasse is reached and the problem solver stops attempting to solve the problem. Incubation can last from a few minutes to many years, during which the attention of the problem solver is not devoted to the problem. Incubation is mostly implicit processing in EII. This is consistent with EII's account of the difference of conscious accessibility between explicit and implicit knowledge.

The third stage, insight, is the “spontaneous” manifestation of the problem and its solution in conscious thought. The spontaneous materialization of a solution is often thought of as a flash of insight or the “Eureka!” moment. In EII, insight is obtained by the process of explicitation, which makes the output available for verbal report. It is worth noting that the subjectively perceived intensity of insight is continuous (Bowers et al., 1990). Correspondingly, explicitation is continuous in the EII theory (using an “internal confidence level” or ICL; Hélie and Sun, 2010). In particular, when the ICL of an output barely crosses the threshold, the output is produced but does not lead to an intense “Aha!” experience. In contrast, when the ICL of an output suddenly becomes very high and crosses the threshold, a very intense experience can result. According to the EII theory, intense insight experiences most likely follow the integration of implicit and explicit knowledge, as it can lead to a sudden large increase of the ICL and synergy.

The fourth stage, verification, is used to ascertain the correctness of the insight solution. Verification is similar to preparation, because it also involves the use of deliberative thinking processes of logic and reasoning. If the verification stage invalidates the solution, the problem solver usually goes back to the first or second stage and this process is repeated. Similar to the preparation stage, verification is accounted for by explicit processing in EII.

While EII is based on Wallas' high level description of creative problem solving, it also captures other theories of creativity, such as the Geneplore model and blind-variation selective-retention (BVSR) theory of creativity (Hélie and Sun, 2010). For instance, as per conscious work theories of creativity, creativity in EII theory can result from explicit processing alone and does not require implicit knowledge to be above an insight threshold.

### Previous work

Explicit-implicit interaction theory integrates existing theories of creativity by detailing the processes involved in key stages of the ideation process. It does so with enough precision to allow implementation using a computer model. A computer implementation of EII theory has been used to account for creativity in several instances (Hélie and Sun, 2010). These include incubation in a lexical decision task, a rare-word association decision task used to test the effect of incubation on the recovery of infrequently used words (Yaniv and Meyer, 1987); incubation in a free-recall task, a retrieval task used to measure the effect of a respite period on the number of new words recalled (Smith and Vela, 1991); and insight in problem solving, e.g., requiring participants to explain why the sight of a shotgun replaces a man's need for a glass of water (Durso et al., 1994). In the first two examples, EII theory accounts for unconscious work that leads to retrieval of distant memories. In the last example, EII accounts for an individual's ability to explore unlikely explanations for a novel situation. Thus, while EII can be purposed to account for various creative problem solving tasks, it is deployed here to replicate and extend remote associates test used to explore the relationship between paradox and creativity.

### Computational model of EII theory

Below the key equation and concepts of the computational model essential for understanding the content of this article is presented. The interested reader is referred to the Appendix of Hélie and Sun (2010) for a complete and detailed mathematical exposition of EII.

The equation of EII theory central to this article is the decision function, which formalizes the output of an idea:

(1) $$\begin{array}{r} \text{P}\left(y_{\left[integrated\right]i}\right)=\frac{e^{y}{\left[integrated\right]}_{i}/\alpha }{\underset{j}{\sum }e^{y{\left[integrated\right]}_{j}/\alpha }} \end{array}$$

where *y*_[*integrated*]*i*_ represents a decision maker's support for idea *i* after integrating the results of explicit and implicit processing and α is the disturbance parameter. The decision function determines an individual's confidence in an idea and the probability that one idea is selected for output over another (Hélie and Sun, 2010). The probability that idea *i* is selected is simply *P*(*y*_[*integrated*]*i*_). Regardless of which idea is selected, confidence in the selected idea, represented by the internal confidence level or *ICL*, is defined as *ICL* = max_*i*_ [*P*(*y*_[*integrated*]*i*_)].

Past simulations of EII have used a higher α to account for more diffuse search in memory (Hélie and Sun, 2010). The transformation represented by Eq. (1) generates normalized activation patterns, with probabilities associated with each idea. Low α levels tend to exaggerate those differences and high α levels tend to reduce those differences. Hence, low α levels are more likely to have a high ICL, which, to re-iterate, is the probability of the most likely idea (i.e., the maximum probability). Output is achieved if the ICL crosses the pre-determined threshold ψ. Resultantly, lower α are related to a greater chance of output.

### Simplified example of computer model

The following simplified example illustrates how the decision function (Eq. 1) operates. In this example the focal agent is assumed to have only two possibilities: idea A and idea B. It is assumed that after the agent has engaged in implicit and explicit processing, her support for idea A (*y*_[*integrated*]*A*_) is 0.60 and her support for idea B (*y*_[*integrated*]*B*_) is 0.40. Support for an idea is the activation strength of a node that represents that particular idea. Activation strength is a function of explicit and implicit processing and represents the “fit” between stimuli (e.g., problems) and ideas (e.g., solutions). In this example, explicit and implicit processing has resulted in a slightly stronger activation of idea A. That is, the agent believes more strongly that idea A is appropriate. However, the ultimate selection of an idea depends not only on the result of explicit and implicit processing, but also on predispositions toward creative thinking, characterized by the willingness to tolerate novel information and the capacity to search in memory for unusual information. These are modeled using ψ and α in EII theory.

Alpha (α) in Eq. (1) can be used to model the capacity to search in memory for new or unusual information. High α levels in the decision function increase the level of “noise” in the model, while low α levels reduce it. For instance, in this example an α value of 1 means the probability (activation strength) that A is selected is 0.55 $\left(P\left(A\right)=\left(\frac{e^{0.6/1}}{e^{0.6/1}+e^{0.4/1}}\right)≅0.55\right)$ and the probability that B is selected is 0.45. The ICL (i.e., *Max*[*y*_[*integrated*]*i*_]) in this example is 0.55 (i.e., *Max*[P(*y*_[*integrated*]*A*_), P(*y*_[*integrated*]*B*_)] = P(*y*_[*integrated*]*A*_)). In contrast, lower α values would decrease noise and increase confidence in an idea $\left(e.g.,\;\alpha =0.1;\;P\left(A\right)=\left(\frac{e^{0.6/0.1}}{e^{0.6/0.1}+e^{0.4/0.1}}\right)\;≅0.88;\;ICL≅0.88\right)$, while higher α values would increase noise and decrease confidence $\left(e.g.,\;\alpha =10;\;P\left(A\right)=\left(\frac{e^{0.6/10}}{e^{0.6/10}+e^{0.4/10}}\right)\;≅0.51;\;ICL≅0.51\right)$. Note that in each scenario, the ICL is not affected by which idea is ultimately selected. Even in cases where the less likely idea is selected, the ICL is still the probability of the most likely idea.

Psi (ψ) is a threshold on the ICL. If the ICL is above ψ, then the selected idea is output to the outside world and processing stops; if the ICL is below ψ, then the idea is kept private and more processing occurs. Hence, ψ can be used to model acceptance of novel information. High ψ values demand a strict preference for an idea before it is output. Hence, if the threshold (ψ) in the current simplified example is 0.6, an idea would be output in the low α scenario (0.88≥0.60), but not in the high α scenario (0.51 < 0.60). If the threshold is changed to 0.4, the ICL would exceed the threshold in the two higher integration scenarios (α = 1 and α = 10), and an idea would be output. The last scenario also demonstrates that when the threshold is low then multiple ideas may cross the output threshold at the same time.

### Modeling integrative complexity

The concept of integrative complexity was developed to capture differences in thinking style—that is, how individuals react to environmental stimuli (Tetlock and Suedfeld, 1988; Tetlock et al., 1993; Suedfeld and Tetlock, 2001; Wong et al., 2011). Therefore, integrative complexity is used to describe an individual's thinking style. Integratively complex thinkers are more likely to generate linkages among disparate concepts and more willing and capable of tolerating different perspectives. In contrast, integratively simple thinkers dislike ambiguity and form dichotomous impressions (e.g., good vs. bad) about people, events, and issues (Schroder et al., 1967). Two cognitive indicators can be used to categorize a thinking style as integratively complex or integratively simple: *evaluative differentiation* and *conceptual integration* (Schroder et al., 1967; Tetlock et al., 1993).

#### Conceptual integration

Conceptual integration, henceforth called integration, is “the capacity and willingness to generate linkages between [ideas]” and “to appreciate interactive patterns of causation” (Tetlock et al., 1993, p. 500). People that score high on integration detect more interactions and connections among points of view. Individuals scoring low on integration fail to appreciate nuances and subtleties, because they fail to see interdependencies among concepts. If integration is the ability to see nuances and subtleties (Tetlock et al., 1993) and if it is associated with a widening of one's search for information (Satish, 1997), than it follows that it can be represented in EII theory by a higher α value in the model. Low noise favors a narrow search and stereotypical responses; in contrast, high noise leads to a more complete and integrated search. As such, a higher α parameter value will increase conceptual integration.

#### Evaluative differentiation

Evaluative differentiation, henceforth called differentiation, entails “the capacity and willingness to tolerate different points of view” (Tetlock et al., 1993, p. 500). Individuals that score high on differentiation actively seek out information about the world and are open to new experiences. In contrast, individuals that score low on differentiation hold contempt for others' points of view and dislike novel stimuli, and are more likely to dismiss these stimuli, than are those that score high on differentiation.

An important aspect of EII theory is that an individual has a subjective confidence evaluation of the appropriateness of each idea. This is denoted by internal confidence level or ICL. If the individual's ICL for an idea crosses the threshold (ψ), insight or the generation of an idea occurs. In other words, insight does not occur if agents are not sufficiently confident in an idea. Because individuals that score high on differentiation are more likely to tolerate points of view they themselves may not believe in or are unsure about, differentiation can be represented in EII using the threshold parameter (ψ). Note that a *lower* ψ is used to represent *higher tolerance* for novel ideas and greater differentiation, so ψ is inversely related to differentiation.

### Experimental setting

While the EII model has been validated over a range of situations (Hélie and Sun, 2010), its validity for describing the effects of paradox on creativity is examined next. To do so, the laboratory experiments in Miron-Spektor et al. (2011) are replicated.

Each of Miron-Spektor et al.'s (2011) four studies consisted of two parts. The first part was a control condition and a priming condition used to expose participants to paradox. The second part was a creativity task used to assess participant creativity. The creativity test in each study was the remote associates test (RAT). The RAT was developed by Martha Mednick (1962) and has since been considered a valid measure of creativity (Fong, 2006; Kaufman et al., 2008). An example of a RAT problem is presenting participant with the cue words “sense, courtesy, place” and asking them to find a fourth word^1^ that is associated with all three cue words.

Studies 1 and 4 used the same manipulation: the reading of a description about a craft product. Although the product was the same in all conditions, several elements of its description were varied to create the treatment condition, which was used to prime the paradox. In study 1, the authors used three different control conditions and one paradox treatment condition; in study 4, the same manipulation was used, but it was varied across conditions in order to prime a low differentiation-low integration condition, a high differentiation-low integration condition, a low differentiation-high integration condition, and a high differentiation-high integration condition. In other words, study 4 primed integration and differentiation independently of one another. In both studies, after reading the description, participants completed the RAT creativity test. In study 1, participants were given 10 RAT problems and had 6 min to complete the test. In study 4, participants were asked to solve the same number of problems in only 4 min.

Studies 2 and 3 used a different manipulation. In these studies, the authors tested whether paradox led to increased creativity when individuals themselves created a paradox, rather than when paradox was activated by specific outside dimensions or criteria. Namely, in part one of these studies, participants were given a “Recall Skill” task, in which they were asked to engage in writing either interesting statements they encountered in the past (control group) or paradoxical statements that they think are interesting (treatment group). In both studies, participants had 4 min to solve as many of 17 RAT problems as they could. Study 3 was used to measure integrative complexity using a Picture Story Exercise (Tetlock et al., 1993) but was otherwise identical in its control and treatment condition to Study 2. Because the simulation does not include perceptual-motor components, the results would not differ between Studies 2 and 3. Therefore only the simulation results of Study 2 are presented.

## Validating the simulation

### Study 1

To simulate the results of Miron-Spektor et al.'s (2011) Study 1, one thousand simulations were run for both the control and treatment conditions. In the control condition, α is set to 550 and a ψ to 0.45. Creative output (i.e., measured as the number of correct solutions in the RAT problems) in the simulated control condition (*M* = 3.87) closely replicated the average of the three control conditions (*M* = 3.88) obtained from human participants. These results support that the α and ψ used in EII account for average level of integrative complexity of the college students in the Miron-Spekter et al. study.

**Figure 2:**
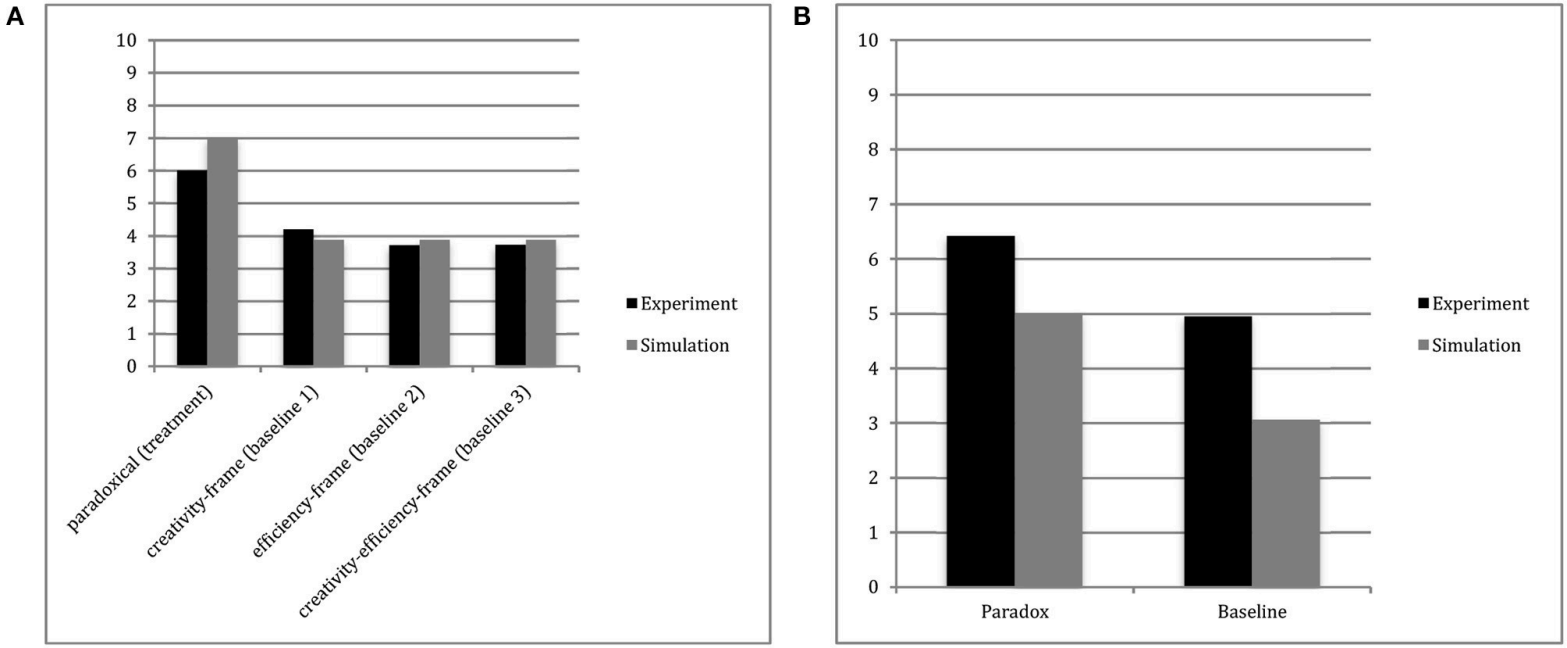
Number of RAT problems correctly solved by condition. Simulated data from EII theory model and human experiment data from Miron-Spektor et al.'s (2011) Study 1 **(A)** and Study 2 **(B)**. The R-squared statistics for the fit between the simulated and human data was 0.997 for Study 1 and 1.000 for Study 2.

To simulate the introduction of the paradoxical tension, a constant of 450 was added to α and a constant of 0.20 was subtracted from ψ. These changes represent the triggering of a more integratively complex thinking style in individuals. Like in Miron-Spektor et al. (2011), the creative output in the simulated treatment condition (*M* = 7.03) was higher than the creative output in the control condition. The results of the simulation are compared with those of the experiment in Figure 2A. The fit between the simulation model and the data was *R*^2^ = 0.997.

### Study 2

To account for the results from Study 2, one thousand simulations for both the control and treatment conditions were ran. To replicate the control the same baseline values were used as in the previous replication, α = 550 and ψ = 0.45. As before, a constant of 450 is added to α and a constant of 0.20 is subtracted from ψ to simulate the paradox treatment. The values for the number of RAT problems to be solved and time constraints on participants were adjusted to match those of Study 2. The results account for those of Miron-Spektor et al. (2011). The paradox group produced more solutions (*M* = 5.01) than did the control group (*M* = 3.06). A comparison of simulated and experimental results can be found in Figure 2B. The fit between the human data and the simulation model was *R*^2^ = 1.000.

### Study 4

Introducing paradoxical tensions increased creativity in the simulations of Studies 1 and 2. Explicit-implicit interaction theory was able to account for the difference in creativity between the control and treatment conditions when the time limit and number of RAT problems were changed. In Study 4, Miron-Spektor et al. (2011) manipulated the baseline levels of integration and differentiation independently. Their study was replicated by adding a constant to α and subtracting one from ψ, independently. One thousand trials were run for each condition. The same baseline levels of α (550) and ψ (0.45) were used for the control group and the same constant change in α (+450) and ψ (−0.20) was applied to replicate the paradox primes.

**Figure 3:**
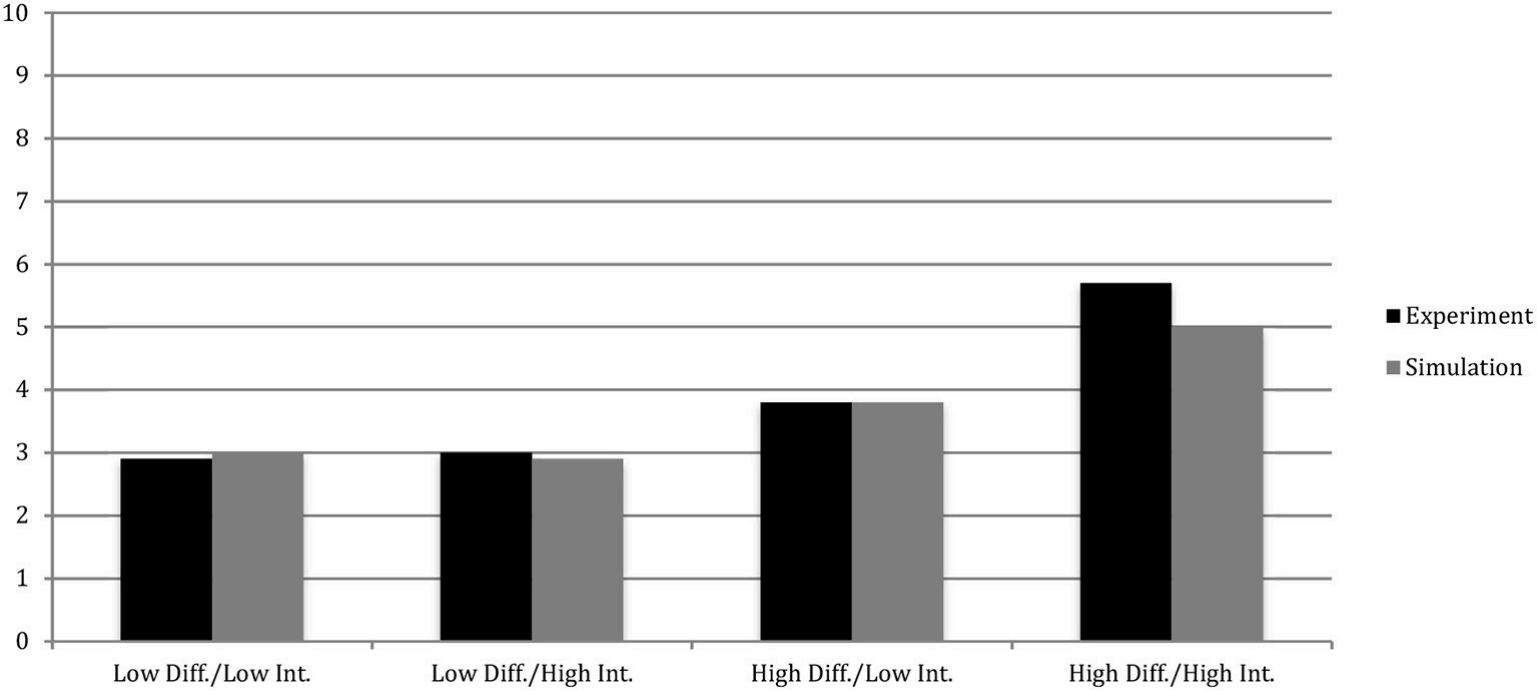
Comparison of correct RAT solutions by condition between simulated results and human experiment data in Study 4 of Miron-Spektor et al. (2011). The R-squared statistics for the fit between the simulated and human data was 0.994 for Study 4.

In the *low*-differentiation-*low* integration condition, simulated agents generated an average of 3.0 solutions, compared to 2.9 by human participants. In the *low* differentiation-*high* integration condition, simulated agents generated 2.9 solutions, whereas human participants generated an average of 3.0. In the *high* differentiation-*low* integration condition, simulated agents discovered an average 3.8 solutions, the same as human participants. In the *high* differentiation-*high* integration condition, simulated agents generated 5.0 solutions, compared to 5.7 by human participants. Like the experimental data, simulation results demonstrate separate effects of differentiation and integration on creative output. Simulation results demonstrate excellent fit with Study 4's human data (*R*^2^ = 0.994). These results are presented in Figure 3.

### Summary

Overall, the results of the EII simulations accounted for the effects of paradoxical frames on creative output found by Miron-Spektor et al. (2011). Specifically, an independent constant change in α and ψ was able to replicate the effect of paradox on creative output found in four laboratory studies. Next, Section Analysis of Thinking Styles explores the central question of this paper, i.e., the differential effect of paradoxical frame on creative output as a function of thinking styles.

## Analysis of thinking styles

This section extends paradox theory by using EII theory to explain and simulate how paradox interacts with an individual's thinking style. First, the effect of diversity in integrative complexity on creative output under situations of paradox is explored. Second, the independent effects of integration and differentiation on creative output, when agents are treated with paradox, is explored.

### Creative responses to paradox

Thinking styles described by integrative complexity are relatively stable. That is, the search for new and novel information (integration) and the tolerance of novel ideas (differentiation) remains unchanged over a wide range of external stimuli (Schroder et al., 1967; Streufert and Nogami, 1989; Tetlock et al., 1993; Satish and Streufert, 1997). There are, however, certain conditions under which an individual's thinking style may become more complex, such as when an individual finds herself subject to accountability pressures (Tetlock, 1983), in multicultural settings (Tadmor et al., 2009), and under paradoxical tensions (Miron-Spektor et al., 2011; Hahn et al., 2014). The more thinking increases in integration, differentiation, or both, the more it is described as being complex.

Many gradations or thinking style levels could be described along the integrative complexity continuum; however, this section focuses on three: low-low, low, high. This section begins with a description of integratively complex individuals as either low or high (Miron-Spektor et al., 2011) on either cognitive indicator of integration or differentiation and follows this by adding a third gradation for each: low-low. Low-low allows for the simulation of a thinking style that is lower on each indicator than the low gradation in Miron-Spektor et al. (2011). The result is three gradations for differentiation and three for integration. “Low” represents the gradation level equivalent to the untreated group in Miron-Spektor et al.; “high” is higher than their untreated group; and “low-low” is lower than the untreated group. These are merely points on a continuous dimension that have been selected for the purposes of communication.

These three gradations are used to construct seven thinking styles, as described by the creative performance of each style (Table 1). An intermediate thinking style, which produces the same levels of creative output as the untreated group in Miron-Spektor et al. and six other thinking styles with varying gradations of integration and differentiation. Cognitive states characterized by strictly opposite levels of integration and differentiation are not described, because such states are unlikely to exist in individuals.

**Table 1:** Creative thinking styles characterized by gradations in integrative complexity.

		**Integration**		
		**Low-Low (**α = **100)**	**Low (**α = **550)**	**High (**α = **1,000)**
**Differentiation**	High (ψ = 0.20)	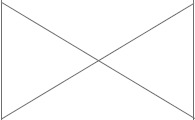	*Receptive* – Increased interest in new information, initial emergence of capacity to combine new information in novel ways; open to new ideas, even those they find distasteful. – High total idea output, both creative and common.	*Complex* – Actively searches for and combines new information; open to new ideas, even those they find distasteful. – High frequency of creative ideas generation and output.
	Low (ψ = 0.45)	*Exploitative* – Recall information in a way that confirms own beliefs; movement away from absolutism, right and wrong are not fixed. – Although creative output is low, certainty in creative approaches is high.	*Intermediate* – Untreated condition in Miron-Spektor et al. (2011).	*Exploratory* – Actively searches for and combines new information; movement away from away from absolutism, right and wrong are not fixed. – High frequency of creative idea generation; large set of creative of creative alternatives results in sensitivity to trade-offs between approaches and lowers creative output.
	Low-Low (ψ = 0.70)	*Simple* – Recall information in a way that confirms own beliefs; compartmentalization of ideas into strict hierarchies. – Relies on well-established way of doing things. Low creative output.	*Rigid* – Increased interest in new information, initial emergence of capacity to combine new information in novel ways; closed to new ideas, compartmentalization of ideas into strict hierarchies. – Strict idea hierarchies inhibit higher levels of creative output.	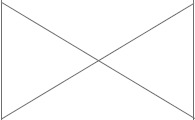

*Baseline simulation parameters for each thinking style are represented by α and ψ values*.

### Diversity in thinking styles and creative output

Creative output is an interactive function of conditional and dispositional factors (for a review, see Anderson et al., 2014). So, how does disposition to a thinking style affect creative output in conditions of paradox? Given the view that the more complex, the better (Perry, 1970; Loevinger and Blasi, 1976; McAdams, 1990), one expects paradox to temporarily increase creative output across the dispositional spectrum. The next section investigates short-term effects of paradox on creativity for each thinking style. First, the baseline or control condition creative output of each thinking style is simulated using the same experimental settings for number of RAT problems (10) and available time (6 min) used to simulate the Miron-Spektor et al. (2011) Study 4. Second, the creative output in paradoxical conditions is simulated, again using the same parameters to simulate the introduction of a paradox (α + 450 and ψ – 0.20). Details about the different parameter values are included in Table 1 and in the description of the thinking styles below.

To anticipate, the theory and findings do not validate the view that paradox increases creative output across the dispositional spectrum (Table 2). Paradox decreased creative output for one thinking style and had varying levels of efficacy among the other styles. Furthermore, baseline creative output was not a good indicator of creative output under paradox, supporting the argument that observed creativity might be misleading when it comes to judging how creatively individuals perform in situations of paradox. Importantly, more cognitive complexity is not always better, so managers should focus on matching thinking style to situation in order to get the most out of cognitive diversity present in their organization.

**Table 2:** Effect of paradox treatment on creative performance for each thinking style.

	**Integration**			
		**Low-Low (**α = **100)**	**Low (**α = **550)**	**High (**α = **1,000)**
**Differentitation**	High (ψ = 0.20)	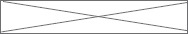	*Receptive* 31.15% increase in creative output.	*Complex* 1.86% increase in creative output
	Low (ψ = 0.45)	*Exploitative* 91.33% increase in creative output	*Intermediate* 72.66% increase in creative output	*Exploratory* 66.44% increase in creative output
	Low-Low (ψ = 0.70)	*Simple* 43.89% increase in creative output	*Rigid* 14.67% decrease in creative output	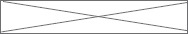

*The introduction of a paradox was simulated by changing the following parameters: α + 450 and ψ −0.20. The percentage increase in creative output is relative to the untreated condition for that thinking style*.

### Simple and complex thinking styles

**Figure 4:**
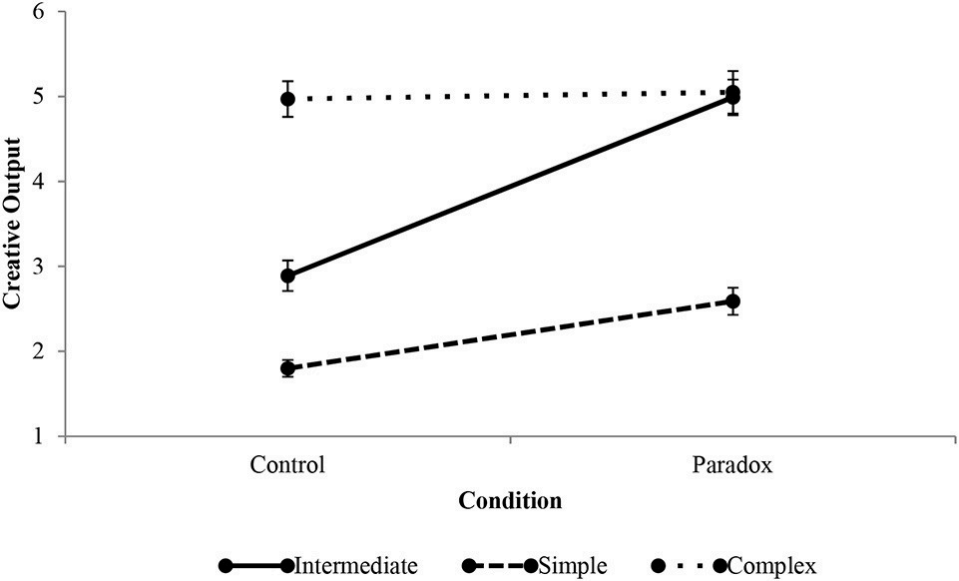
Effect of paradox treatment on intermediate, simple, and complex thinking styles. Error bars indicate standard errors.

Figure 4 graphically presents the differences in creative output between agents that can be described in terms of intermediate, simple, and complex thinking styles (the diagonal in Tables 1, 2). Intermediate and simple thinkers' benefited more from the paradox treatment than did complex thinkers. An important outcome of the simulations is the non-monotonicity of the relationship (slope) between paradox and creative output. The results suggest that the effectiveness of paradox increases with integrative complexity, but only up to a point, after which it plateaus. This implies a non-linear relationship between the thinking style-paradox interaction and creative output. It is also noteworthy that in conditions characterized by paradox, intermediate thinkers perform just as well as do complex thinkers. This suggests that if paradoxical tensions are present, complex thinkers may seem to others to underperform expectations on creativity tasks, particularly compared to otherwise moderately creative performers. Furthermore, simple thinkers may exceed expectations on creativity tasks in paradoxical situations. Below, each profile is described in more detail.

#### Intermediate

The intermediate thinking style was modeled to replicate the creative output of the untreated group in Miron-Spektor et al.'s (2011) Study 4. As a result, it represents a thinking style characteristic of the average college student in their study. This thinking style serves to hold the middle ground around which more and less integratively complex thinkers were modeled. In 1,000 trials the intermediate profile generated an average of 2.9 solutions. Administering the paradoxical tension treatment enhanced creative output by 72.41% over the untreated condition for that thinking style (baseline) to an average of 5.0 solutions.

#### Simple

The simple thinking style is characterized by a dislike for novelty and narrow search for and capacity to combine new information. Simple thinkers are low-low on both indicators of integrative complexity. Therefore, they represent the least integratively complex thinker that was modeled. The simple thinker generated fewer outputs than did the intermediate thinker, with an average of 1.80 solutions over 1,000 simulation runs. To simulate agents using a simple thinking style integration (α = 100) and differentiation (ψ = 0.70) were reduced to a low-low gradation. This allowed for modeling a lower integrative complexity on both cognitive indicators than the untreated group in the Miron-Spektor et al. studies. An individual that can be characterized in terms of a simple thinking style appears to others to be behaviorally deterministic (Driver and Streufert, 1969; Tetlock et al., 1993; Suedfeld and Tetlock, 2001). Behavioral determinism of this profile is not only a function of dislike for the unusual, but also the inability to generate creative output using existing knowledge.

Introducing paradoxical tensions enhanced the creative output of simple thinkers, but this effect was lower than it was for intermediate thinkers. In 1,000 trials, paradox increased creative output to 2.59 ideas, an increase of 43.89% over the control condition. Simple thinkers did not reach the creative output of intermediately complex thinkers and the magnitude of the treatment effect was lower on the creative output of simple thinkers. The difference of change in output between the two styles can be explained by the increasing marginal effectiveness of paradox on more complex thinkers. Although simple thinkers did not outperform their more complex counterparts in paradoxical conditions, they nevertheless benefited from paradox. These results challenge the proposition that cognitive complexity is a necessary condition for superior performance in paradoxical conditions (Smith and Lewis, 2011).

It is important to note that while the simple thinking style may not be best suited to creative work, simple thinkers confidence in ideas was relatively high with an ICL score of 0.61. This was only lower than the ICL of the exploitative thinker who scored 0.85. These findings suggest that simple thinkers are action oriented and may exhibit greater *behavioral complexity*, which connotes action as well as cognition (Denison et al., 1995), than other more cognitively complex styles. Hence, simple thinkers are more likely to output creative ideas, or output them more quickly, than are other thinking styles. The action (i.e., output) orientation of simpler thinking styles is supported by recent work on the differences between managers adopting business case frames and paradoxical frames when responding to sustainability issues (Hahn et al., 2014). Although business case frames result in a simpler thinking style than do paradoxical frames, managers adopting a business case frame focus more on an active approach whereas those adopting a paradoxical frame are more likely to feel ambivalent about a certain approach (Hahn et al., 2014). Moreover, while agents characterized by a simple thinking style were relatively uncreative in control conditions, they nevertheless benefited from the paradox treatment.

#### Complex

A complex thinking style can be described in terms of both high differentiation and high integration. A complex agent has the most integratively complex thinking style that was simulated. The complex style was simulated by increasing α to 1,000 and decreasing ψ to 0.20. This represented a baseline that is higher in both indicators of integrative complexity than the untreated group in the Miron-Spektor et al. (2011) studies. In 1,000 trials agents generated an average of 4.99 solutions. While a simple thinker appears deterministic, a complex thinker appears unpredictable (Tetlock et al., 1993). The ability to perceive the same information in multiple ways allows complex thinkers to have a broad range of responses. This abstract orientation generates a high number of creative outputs and is, as such, highly effective at adapting to changing situations.

The treatment increased creative output to an average of 5.05 solutions, a small increase of 1.86% in creative output over the control condition. Similar to the simple thinking style, the increase in creative output was lower than it was for intermediate thinkers. These results contradict those of previous work that suggests that the benefits of paradox are most likely to accrue to complex thinkers (Smith and Lewis, 2011). The model suggests that complex thinkers are unlikely to be more creative in paradoxical situations than they are in non-paradoxical ones. Conceptually, complex thinkers are already comfortable with novelty and already actively seek out and combine new information. Said differently, complex thinkers may already process information in a manner analogous to working through paradox. As such, paradoxical tensions do not further enhance the creative output of complex thinkers.

### Exploitative and exploratory thinking styles

**Figure 5:**
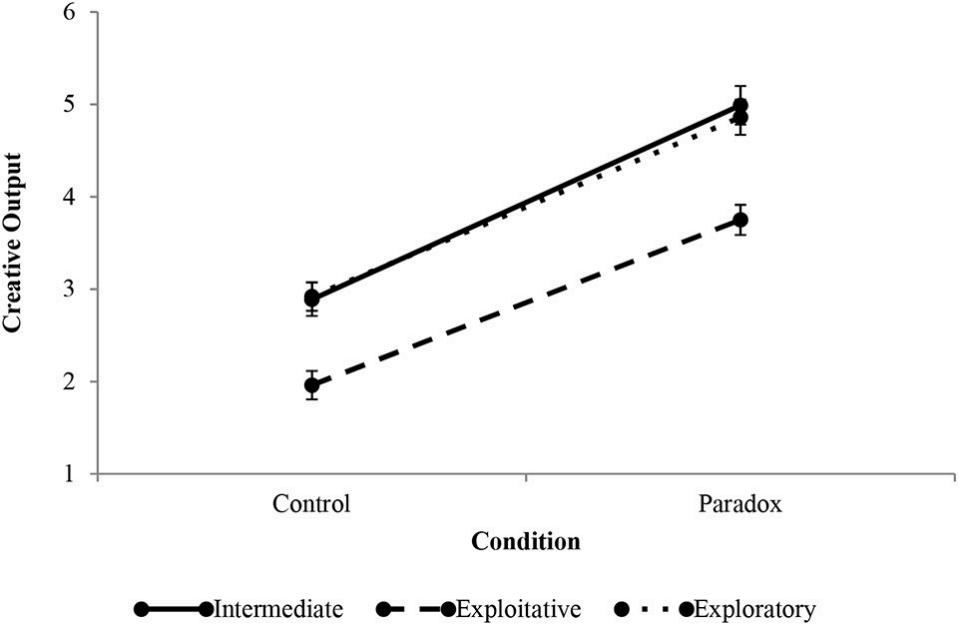
Effect of paradox treatment on intermediate, exploitative, and exploratory thinking styles. Error bars indicate standard errors.

Figure 5 shows the differences in creative output among agents that can be described in terms of intermediate, exploitative, and exploratory thinking styles (low differentiation, middle row of Tables 1, 2). The exploitative profile benefited most from paradox, despite baseline low-low integration. Conversely, exploratory thinkers, who had a baseline of high integration did not benefit from paradox to the same extent. Their post-treatment creative output was not different than that of the intermediate style.

It is worth noting that exploitative thinkers are *less* integratively complex than intermediate and exploratory thinkers, yet they benefited more from paradox than either of these styles. Although their overall creative output fell below that of their more integratively complex counterparts, as one would expect, the simulation predicts that some integratively simpler thinkers may have greater creative potential in paradoxical conditions than previously thought. The practical implications of these findings are discussed in the closing section of this article.

#### Exploitative

The exploitative thinker shares the low differentiation level with the intermediate profile, but is found on the low-low end of the integration spectrum. Individuals characterized in terms of an exploitative thinking style display lower creative output in control conditions than do intermediate thinkers. To simulate the exploitative thinking style, integration was reduced below the untreated group in Miron-Spektor et al.'s (2011)Study 4. Differentiation, however, was simulated at the untreated level in their study. In 1,000 trials, exploitative thinkers generated an average of 1.96 solutions. The key process-based difference between the exploitative and intermediate thinkers is the level of integration. The result is that exploitative thinkers are less willing and capable to search for new information than are intermediate thinkers. Less access to new information reduced the likelihood that novel ideas are generated. Conceptually, fewer generated ideas leads exploiters to perceive fewer alternatives and, as such, have higher overall confidence in decision that are generated (Schroder et al., 1967). The computational outcome of higher internal confidence is that ideas are frequently output—creative or not. Correspondingly, in EII theory, the ICL of exploitative thinkers can lead to intense “Aha!” experiences.

Paradox was especially effective at increasing creative output of the exploitative thinker. One thousand trials of exploitative agents treated with a paradoxical tension condition are simulated. Creative output in the treatment condition increased to an average of 3.75 solutions, an increase of 91.33% over the control condition, the highest increase in creative output of the modeled thinking styles.

The central feature of exploitative thinkers is an excessive confidence in one's point of view. In EII theory, the high level of internal confidence means agents have latent potential to be creative. The latent potential exists because of a capacity to output ideas frequently, and although not all of these ideas are creative, high confidence guarantees that when a creative idea is generated, it is output. Earlier studies on the bias against creativity corroborate these findings. People hold a bias against creativity because they experience a motivation to reduce uncertainty in decision making (Mueller et al., 2012). They are thus less likely to output creative ideas, which are by definition new and thus their outcomes are less predictable than those of conventional and old ideas. In EII theory, uncertainty in ideas is the inverse of internal confidence. Therefore, the higher an agent's ICL, the lower the agent's uncertainty about a decision. These findings support the case for understanding differences in cognitive profiles for scholars of paradox theory. The results suggest that paradoxical situations increase creative output most when they exploit an individual's predisposition to be decisive, which is a thinking style characteristic that is not normally associated with high levels of creative output in stable, non-paradoxical, conditions (Schroder et al., 1967; Tetlock et al., 1993; Satish, 1997).

#### Exploratory

Unlike the exploitative thinker, the exploratory thinker actively searches for and combines new information. While the exploratory thinker also shares the low differentiation level with the intermediate thinker, explorers have a high integration gradation. High integration allows the explorers to discover and combine new information, which exposes them to a large set of alternatives. In 1,000 trials, exploratory thinkers generated an average of 2.92 solutions. In EII, exploratory thinkers display creative output similar to that of intermediate thinkers. The explorer has one apparent advantage over the intermediate thinker—he searches more broadly in memory for information. The advantage of broad search is the generation of more alternatives. According to EII theory, although more alternatives may lead to higher generation of creative ideas they must not lead to more creative output. A large set of alternatives will reduce certainty that one idea is superior to all others and may preclude insight, which is the output or externalization of ideas.

Explorers' average level of confidence was 0.06 above the threshold (ψ). For comparison, intermediate thinkers' internal confidence is on average 0.16 above the threshold, and that for exploitative thinkers 0.40 above the threshold. As previously suggested for explorers, this leads to generated but not output ideas. From an observer's point of view, the exploratory thinker may appear to often agonize over decisions and be biased against creativity (Mueller et al., 2012).

In 1,000 paradoxical condition simulations, exploratory thinkers output an average of 4.86 solutions, a 66.44% increase in creative output from the control condition. Exploratory thinkers represent the dispositional opposite of exploitative thinkers on the integration dimension. As such, they may benefit from working through paradox in different ways. Exploiters do not search broadly for new information and explorers are indecisive about ideas. Whereas exploiters' creative output benefited from higher integration levels resulting from paradox, explorers benefit from high differentiation. Higher differentiation enhanced tolerance for novel ideas and improved creative output. Post treatment, internal confidence was 0.14 above threshold in treatment conditions, a 129% increase in tolerance for novelty over the control condition. Moreover, whereas in the control condition explorers generated (but did not output) an average of 9.94 ideas, only 2.57 were generated but not output in the treatment condition.

### Rigid and receptive thinking styles

**Figure 6:**
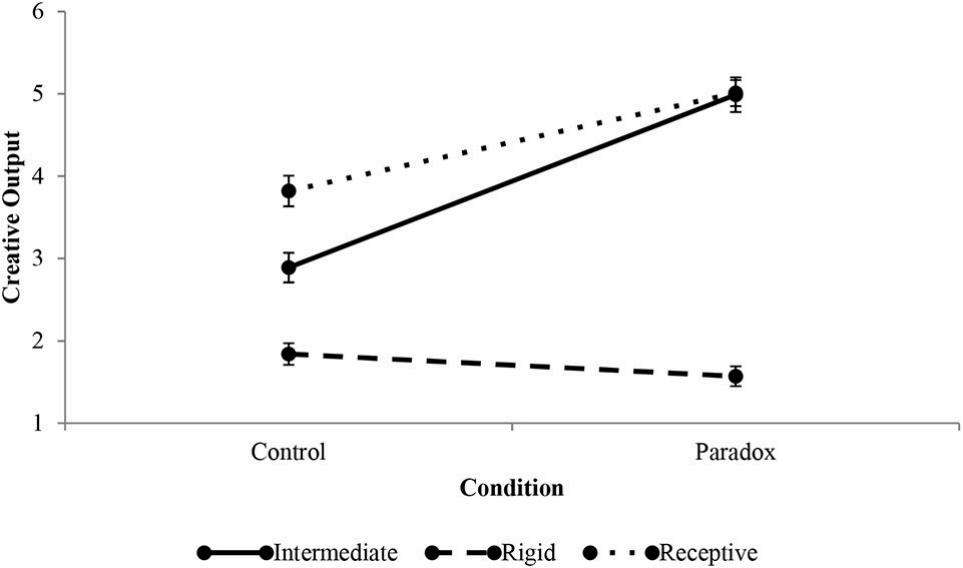
Effect of paradox treatment on intermediate, rigid, and receptive thinking styles. Error bars indicate standard errors.

Figure 6 graphically depicts the differences in performance between a control and treatment condition for agents that can be described in terms of an intermediate, rigid, and receptive thinking styles (low integration, middle column in Tables 1, 2). Two outcomes highlight important predictions of the simulation. The first is lower than expected creative output for the receptive style. Receptive thinkers can be described as more complex on the differentiation dimension than intermediate thinkers. Receptive thinkers outperform intermediate thinkers in control conditions. Yet, under paradox conditions, their performance was not any higher than that of the intermediate profile. Receptive thinkers were open to new ideas, but the openness was underutilized because receptive thinkers did not output any more solutions to RAT problems than did intermediate thinkers. The other important prediction is the decrease of creative output of rigid thinkers. According to EII, lower than intermediate starting differentiation precluded simulated agents from outputting ideas. Even when ideas are generated they are not output, they are kept private.

#### Rigid

A defining characteristic of the rigid thinking style is a strong aversion to novelty and moderate willingness and capacity to seek out new information. Rigid thinkers share the low integration with intermediate thinkers, but have lower differentiation. This means rigid thinkers reject novel ideas that they discover. In 1,000 trials, the rigid thinking style output an average of 1.84 solutions, just below the creative output of the intermediate style. For this thinking style, conventional ideas are seen as superior and considered to have very weak, if any, alternatives. From an observer's perspective, the rigid thinker appears to use simple rules of thumb to decide among a set of complex alternatives.

In 1,000 paradoxical treatment simulations rigid thinkers output an average of 1.57 solutions, a decrease of 14.67% over the control condition (Figure 6). Agents with a predisposition to favor existing ideas, despite some capacity to seek out and combine new information, are more susceptible to indecision than are other thinkers that were modeled. Conceptually, a rigid thinker has the capacity to seek out new information, but is beholden to old ways of doing things. Subjecting this thinking style with the same paradox treatment that was used for the other profiles increased the generation of novel ideas, but the starting low-low gradation precluded rigid agents from outputting these ideas. For rigid thinkers to benefit from paradox, the paradox would need to affect thinking style differently than it did in this simulation. Specifically, greater increases in differentiation, with the same or lower increases in integration, would be necessary to improve creative output.

These findings highlight the importance of how individuals perceive paradox to the study of paradox in general and the study of paradox and creativity in particular. To study the average effect of paradox on individuals, the effect of paradox must be held constant across individuals. Should perception of a paradoxical condition vary between individuals, the outcomes of the study may be confounded by individual experiences. This is especially true in multicultural settings, where perception of what is and what is not a paradox may depend on cultural background (Keller and Wen, unpublished manuscript). In such settings, scholars should expose participants to different paradoxes, as a function of culture, in order to achieve the same treatment effect. This would require ex-ante exploration of how individuals define paradox in a particular culture. For an example of early work, see Keller and Chen (2017).

#### Receptive

High willingness and tolerance for novelty is an important implication of a receptive thinking style. Whereas this profile shares the low integration with the intermediate profile it is characterized with high differentiation. High differentiation permits openness to alternative points of view, even those the individual may find distasteful (Tetlock et al., 1993). After 1,000 trials, receptive thinkers output an average of 3.82 solutions. A person who is functioning at this level is capable of simultaneously seeing the validity of multiple ideas and selecting the most creative one for output. To an observer, a receptive thinker may appear as open-minded.

The paradox treatment increased creative output by 31.15%, to an average of 5.01 solutions. In EII theory, a low threshold represents a receptive thinker. A low threshold benefits especially from higher integration. Introducing paradox creates a more diffuse search in memory, which is likely to enhance creative output. Coupled with tolerance for new perspectives, the interaction between paradox and this thinking style lead to higher creative output.

### Modeling a broader differentiation and integration spectrum

Many gradations of differentiation and integration are possible. An individual may not neatly fit into any of the proposed thinking style categories. She may instead find herself anywhere along the differentiation and integration complexity spectrum. Modeling creative output and the effects of paradox along the complexity continuum may reveal interactive patterns not obvious in the simulations of the above seven styles. To model a broader spectrum of thinking styles, 1,000 simulation trials were run for each 0.01 gradation of ψ, from 0.00 to 1.00. For comparison, this was done for both the control and treatment levels of integration.

**Figure 7:**
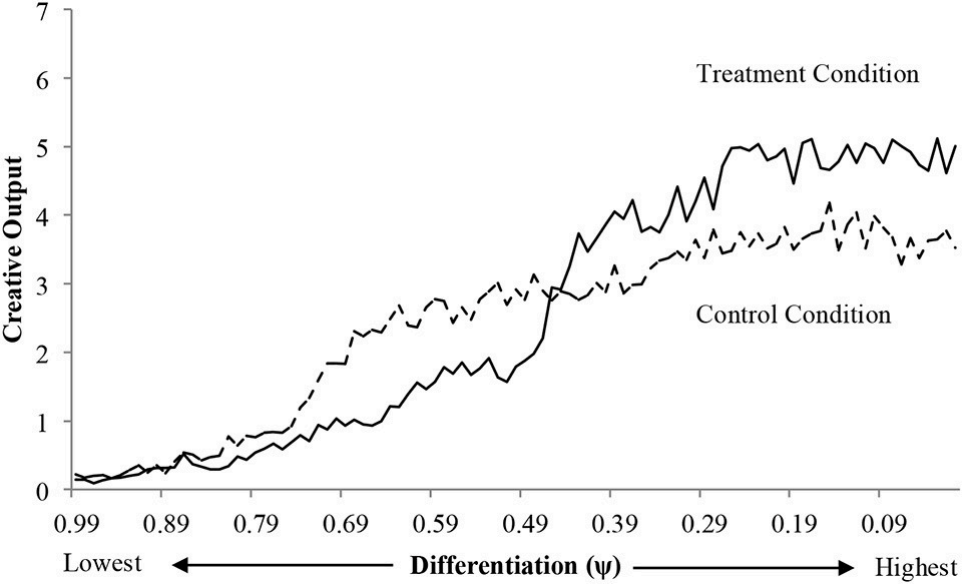
Comparison of creative output between control (α = 550) and treatment (α = 1,000) levels of integration across the differentiation continuum.

The results of the simulation, presented in Figure 7, reveal that the positive effect of paradox on creative output is conditional on a minimal level of differentiation. Below this minimal level of differentiation, the paradox reduced creative output. The rigid thinking style is an example of the potentially detrimental effect of paradox on creative output. For more differentiated thinking styles, paradox should increase creative performance. The workplace implication is careful consideration of how paradox is perceived by rigid thinkers and supports a dynamic decision-making model for managing paradoxes. In line with Smith (2014 p. 1593), the simulation results suggest that the most effective way of engaging paradoxes may be “through a pattern of iteratively choosing between domains over times,” in effect selecting domains that are most conducive to creative performance as a function of thinking style.

**Figure 8:**
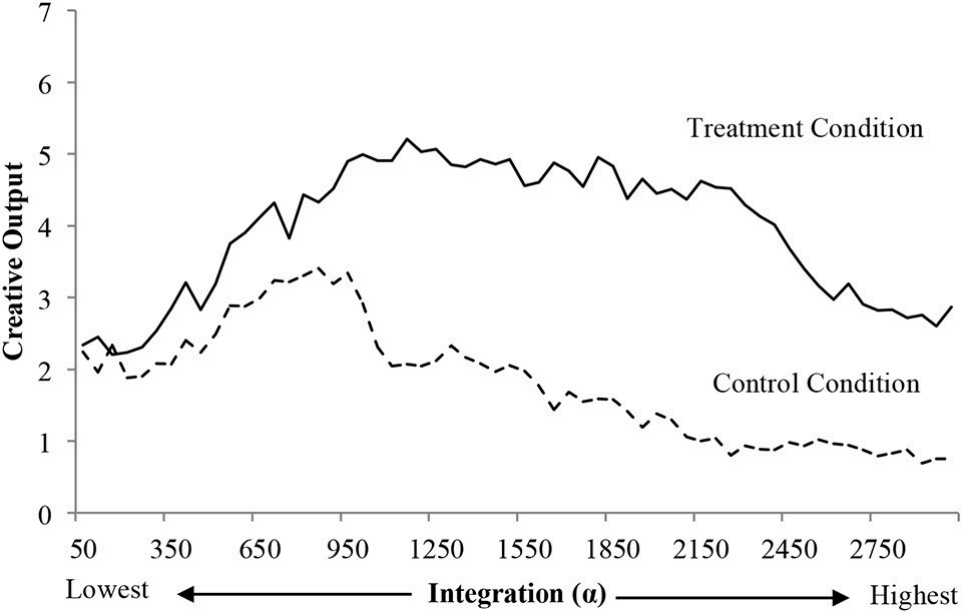
Comparison of creative output between control (ψ = 0.45) and treatment (ψ = 0.25) levels of differentiation across the integration continuum.

The observed limited effectiveness of paradox to increase creative output of explorers and complex thinkers suggests the presence of a ceiling effect. This effect was further explored by modeling thinking style along the integration continuum. Control and treatment conditions were simulated for each integration gradation of 50, from an α of 50 to an α of 3,000. The simulation results reveal a parabolic relationship between integration and creative output (Figure 8). In addition to confirming a ceiling effect of paradox, the parabolic relationship suggests that integration can, after some point, reduce creative output. Increases in integration increase the presence of abstract properties heightening uncertainty. Schroder et al. (1967, p. 21) describe this increase in abstractness as “the sense that alternatives exist,” “not … the sense that the world is more chaotic.” The sense of more alternatives requires much more search for information before resolutions are made and can, given limited time to search for information, result in lower creative output. In EII theory, this means that the idea is kept private and more processing occurs.

**Figure 9:**
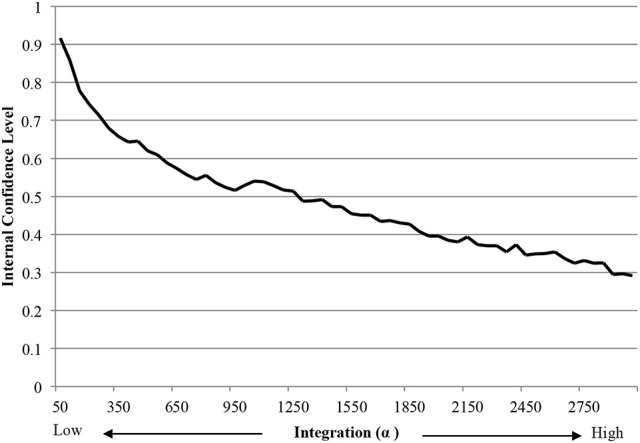
Effect of integration on internal confidence level of simulated agents.

The results also corroborate findings about the bias against creativity. Mueller et al.'s (2012) findings that people reject creative ideas when they experience a motivation to reduce uncertainty reflects the simulation results. Specifically, increasing integration decreased internal confidence in ideas (Figure 9) which, in EII theory, is the equivalent of increasing uncertainty. Together with the parabolic effect of integration on creative output, these findings suggest that paradoxes may interact with thinking style to exasperate the bias against creativity.

## General discussion

This article contributes to the existing creativity and paradox literatures with new findings about individual differences in creative output to paradoxical tensions. Creative output depends on an interaction between situation, paradox, and cognition. A better understanding of thinking styles in the workplace can result in a high organizational creativity, which is correlated with innovation and organizational success (Hennessey and Amabile, 2010; Amabile, 2014; Anderson et al., 2014). This is timely because, as the world becomes more global, fast paced, and competitive, creative output becomes more important to superior organizational and entrepreneurial performance (IBM 2010 Global CEO Study, 2010).

While there is extensive evidence on the *average* positive effect of paradox on creativity in organizations, the key problem of understanding whether all individuals react the same to paradox remained unexplored. This challenge was addressed by simulating the effect of paradox on a range of thinking styles. Findings revealed that stable cognitive differences matter, and that the relationship between paradox and creative output is not simple. Results suggest that individuals of intermediate integrative complexity perform at least as well at creative tasks in situations or paradox as do integratively complex individuals. Indeed, integratively simple thinkers can benefit from paradox. The creative output enhancing effect of paradox was most positive for individuals that are otherwise not creative, modeled as exploitative and intermediate thinkers in the simulations. Although paradox had a negative effect on the creative output of individuals that can be described as rigid thinkers, the results suggest that strategically, and potentially iteratively, choosing between domains can increase creative output even for rigid thinkers. Whenever faced with paradoxical tensions, managers should not necessarily look for new and original solutions from creative organizational members. Rather, strategic management of the work environment based on thinking style will lead to the most effective utilization of creative resources to maximize creative output. In fact, the results suggest that if manager seek to increase organizational member creative output in situations of paradox, they should look to individuals of otherwise average creativity.

Managing paradox for creativity involves a deep understanding of the interactions between situational and individual level variables. Indeed, embracing paradox may reduce creative output in some cases. Even still, creativity need not be desirable. The generation and output of creative ideas does not guarantee their economic or social value. Thomas Edison holds the record for the most patents awarded to a single person by the US Patent office. As pointed out by Simonton (1997), not all of these patents turned out to be profitable. As it happens, the cost of one of these patents exceeded Edison's profits for the electric light bulb. Although beyond the scope of this study, this reasoning implies the need for a further discussion about the economic value of creativity, and therefore the economic performance implications of creativity resulting from paradox. Good managerial judgment is necessary when deciding under which circumstances creative output will strengthen competitive advantage.

Creativity also requires action. In the entrepreneurial sense (Schumpeter, 1928, 1942), for creative output to be meaningful, it must be about more than just discovering new ideas. It also requires aggressive, bold, and confident action on those ideas (Kirzner, 1999; McMullen and Shepherd, 2006). How paradox affects action on creative ideas is only suggested in this study, but it should be an important avenue for future research. Although successful introduction of ideas is difficult to measure, EII theory can provide a sense of when agents are more likely to act. Agents with higher confidence in their ideas are also more likely to act on them. More confident people are more likely to feel an intense “Aha!” experience and may take the risks necessary for innovation. Still, more work is needed to relate idea generation to action, and simulation models can be useful in generating testable predictions.

### Assessment of individual differences in integrative complexity

Of course, simulations cannot replace laboratory experiments. The purpose of simulation is to generate hypotheses to guide laboratory experiments. As such, future studies should attempt to confirm the findings using human subjects. In this article, the cognitive indicators of integration and differentiation were chosen for simulation as test materials and questioners for assessing integrative complexity are readily available and validated. As such, these procedures can be used to categorize participants within the thinking styles presented here. The available procedures for assessing integrative complexity were originally developed by Schroder et al. (1967) and later refined by Tetlock and Suedfeld (1988).

The two cognitive stylistic variables of differentiation (i.e., capacity and willingness to tolerate conflicting interpretation) and integration (i.e., development of conceptual connections) are rated on a 1–7 scale, in which scores of 1 signify low levels of both integration and differentiation and scores of 7 indicate high scores on both dimensions. Assessment proceeds with trained integrative complexity coders independently assigning complexity ratings to responses of pictorial stimuli used in a picture story exercise (PSE). Stimuli include pictures of a man and a woman sitting together at a table in a restaurant, a picture of a worker at his desk, a picture of two female scientists at work in a laboratory, a picture of a male ship's officer speaking to someone else not in uniform, and so on.

Prototypical responses of 1 on both integrative complexity responses only consider one point of view of what is happening in the picture (only one of the subjects) and dichotomize potential outcomes (a clear way to proceed). On the other hand, prototypical responses of 7 show evidence of different points of views. For instance, responses that rate high in their response to the two female scientists propose different theoretical points of view on viral reproduction of the two scientists and recognize the difficulty in integrating these two points of view into a more general integrative formulation. Other assessments report significant correlations with integrative complexity and could be used for broad categorization of participants into the above developed creative thinking styles.

Tetlock et al. (1993) present a number of correlations of integrative complexity with other measures. The Myers-Briggs Type Indicator (MBTI) scales of Intuition (*r* = 0.41; *p* = 0.001) and Perception (*r* = 0.56; *p* = 0.001) correlate highly with integrative complexity. The strongest correlations are with the Adjective Check List Creative Personality (*r* = 0.27; *p* = 0.004) and Free Child (*r* = 0.20; *p* = 0.04) scales. The California Psychological Inventory (CPI) sees a number of significant correlations with integrative complexity: Creativity (*r* = 0.27; *p* = 0.004), Independence (*r* = 0.21; *p* = 0.03), Flexibility (*r* = 0.19; *p* = 0.04), Empathy (*r* = 0.19; *p* = 0.04). Hence, the simulation results presented here can be compared with results obtained through studies conducted with human participants.

The results of this study are complementary to human studies, as we build new theory about the private cognitive mechanism responsible for the effects of paradox on creative outcomes. While beyond the scope of this study, it would be interesting to explore whether the relationships developed here using integrative complexity hold true for other personality measures—such as those that Tetlock et al. (1993) find correlate highly with integrative complexity. For example, one could replicate the study or Miron-Spektor et al. (2011) while administering one (or several) of the aforementioned assessment methods and explore how the introduction of a paradox differently affects participants as a function of the results in the assessment tests. This experiment would allow for beginning to test empirically the predictions made by the presented simulation work.

### Limitations and future research

Like in all other simulation studies, operationalizing the complexity of human cognition using a computer is limited by the assumptions of the cognitive architecture. Nevertheless, the use of simulations allows for new insights and has therefore been encouraged in psychology (Besold et al., 2015) and organizational science (Gavetti et al., 2007; Powell et al., 2011). In this article, we utilized a computational model validated in cognitive science to address empirically challenging phenomena. This simulation allowed for observing the effects of paradox on a range of thinking styles. The simulation was validated specifically for the study of paradox and creativity by accounting for data from previously published work (Miron-Spektor et al., 2011). The results reveal an interesting and, as of yet, empirically unobserved relationship, thus providing a new perspective on an existing theory and generating new predictions.

In order to expand existing theory, the empirical creativity tests used in those studies was simulated. These approaches are based on the remote associates test (RAT) of creativity, which is just one test of creative problem solving. Future research should attempt to replicate the simulation results using other creativity tests—such as the alternative uses test (Wilson et al., 1953) or creative writing (Davis and Subkoviak, 1975). Furthermore, the RAT relies on insight—the output of an idea. However, creative problem solving must not produce insight. Future research should test existing theories without explicitly relying on creative output as a measure of creative ideation.

Future research on paradox and creativity would benefit from field observations of how paradox affects creative performance—especially over different contexts of time and space. Such studies would require the classification of paradox as well as measurements of individual levels of integrative complexity. Future research should also address the effects of paradox at different levels of analysis, such as the team level. Scholarship in this direction could provide significant insights into organizational and workplace design, strategic reactions to contradictory demands, and hiring decisions.

Another limitation is that the work in this article was based on previous research findings that paradoxes increase integration and differentiation, which increase creativity. With the discovery of a more fine-grained relationship between paradox and creative output, a more fine-grained inquiry into paradox is also warranted. Not all paradoxes will likely be alike in intensity, relevance, and salience to a given decision maker. Future work should attempt to distinguish between different types of paradox, such as social and business tensions (Gonin et al., 2013). While the current study focuses on refining the picture of how paradoxes may *in general* influence creativity, similar work remains to be done on the effects of *specific* paradoxes on creativity.

## Author contributions

Both authors designed the experiment and hypotheses, and interpreted the results. GC wrote the code, performed the simulations and analyses. The manuscript was written by both authors.

### Conflict of interest statement

The authors declare that the research was conducted in the absence of any commercial or financial relationships that could be construed as a potential conflict of interest.

## Footnotes

^1^The answer is “common”.
